# A qualitative study of public health nurses' perspectives and experiences on nutritional guidance for parents of infants and toddlers

**DOI:** 10.1111/mcn.13546

**Published:** 2023-07-13

**Authors:** Christine Helle, Elisabet R. Hillesund, Nina Cecilie Øverby

**Affiliations:** ^1^ Department of Nutrition and Public Health, Faculty of Health and Sport Sciences University of Agder Kristiansan Norway

**Keywords:** child public health, counselling, infant and child nutrition, nurse, parents, qualitative methods

## Abstract

In Norway, public health nurses (PHNs) are responsible for giving parents nutritional knowledge, but limited research describes how they perceive this task. This study explores PHNs' perceptions and experiences on nutritional guidance for parents of infants and toddlers. Semistructured interviews with six PHNs were conducted and transcribed verbatim. Data were subjected to thematic analysis. Five main themes were identified: (1) Dietary guidance for parents is central to the work; (2) PHNs perceive they have parents' trust, and parents are in general open to nutrition counselling; (3) food and meals must be seen in light of the family context; (4) The dialogue must be adapted to the individual family; and (5) PHNs have expertise on nutrition; however, updating knowledge is difficult. Nutritional guidance was perceived by PHNs as a core activity. They felt that they had parents' trust, and that parents were particularly open to nutritional guidance during the first 2 years. Counselling was generally well received, but conversations on overweight were perceived as difficult. PHNs strove to tailor their guidance to individual needs. However, providing guidance on a wide range of issues in different families and cultures could be challenging. They acknowledged a need for updating knowledge but the offer of courses was sparse. Our findings suggest a discrepancy between how nutrition is prioritized in the education of PHNs and what they encounter in clinical practice. In the future, this should be given more attention given the PHNs' unique position to promote healthy eating and long‐term health.

## Key messages

- Guidance on diet and nutrition is perceived by public health nurses as a core activity and constitutes a large part of their communication with parents of infants and toddlers.
- Public health nurses strive to tailor their counselling to meet individual and cultural demands. Conversations around childhood overweight and obesity are perceived as sensitive and difficult.
- Counselling parents on children's healthy diet places great demands on nutritional knowledge and communication skills. There seems to be a discrepancy between how nutrition is prioritized in the education of public health nurses and what they encounter in clinical practice.

## Graphical abstract

In Norway, public health nurses (PHNs) are responsible for giving parents nutritional knowledge, but limited research describes how they perceive this task. This study explores PHNs' perceptions and experiences on nutritional guidance for parents of infants and toddlers. Dietary guidance for parents was perceived as central to the work of PHNs, but our findings suggest a discrepancy between how nutrition is prioritized in the education of PHNs and what they encounter in clinical practice

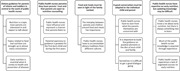

## 1 INTRODUCTION

The first 1000 days of life, from conception until the child's second birthday, is a window of opportunity for long‐term health and well‐being (Schwarzenberg et al., 2018). Failure to meet nutritional needs in this early stage of life is strongly linked to lifelong risk of obesity and non‐communicable diseases (NCDs) (Hanson & Gluckman, 2014). The relevance of the first 1000 days from a public health perspective is supported by cost–benefit analyses showing large economic and social returns from early‐life investment in general, especially from optimizing nutrition (Campbell et al., 2014). In the July 2020 *Lancet* report, United Nations leaders called for action on maternal and child nutrition, as nutritional factors in early life have a crucial role in determining health and disease throughout life (Clark et al., 2020). The World Health Organization (WHO) promotes a life‐course approach to target health and health inequality (WHO, 2018). This entails to positively influence nutrition and diet during sensitive dietary transitions, such as the start of breastfeeding or the introduction of solid foods (Darnton‐Hill et al., 2004).

In Norway, these dietary transitions are addressed through public health nurses (PHNs) working at municipal child health centres (CHCs). All Norwegian children from birth to 5 years of age, are regularly seen at CHCs, where the role of the PHNs is specifically aimed at health promotion and illness prevention (Clancy, 2007). The CHCs are required to follow the standards for investigation, treatment and follow‐up as presented in the Norwegian official recommendations for the service (Norwegian Health Directorate, 2017). According to these guidelines, topics related to nutrition are to be addressed in every consultation during the child's first 4 years of life.

Despite the important task laid upon the PHNs in terms of early dietary guidance, there is limited research on how PHNs perceive this work. A Finnish study (2012) reported data from 327 PHNs. They perceived nutrition counselling as an important but challenging task, especially related to the large variety of issues they had to discuss. They suggested that more time and updated educational resources would improve their work (Ilmonen et al., 2012). A Norwegian study of nutrition counselling towards immigrant mothers found similar results (Holmberg Fagerlund et al., 2016). In a more recent Swedish study, the PHNs reported an increased need for support among today's parents on lifestyle issues, including nutrition‐related topics (Ersson et al., 2021).

The aim of the current study was to broadly explore the PHNs' perspectives and experiences on nutritional guidance for parents of infants and toddlers. We wanted to identify potential facilitators, barriers and needs to provide good guidance and appropriate care that could be useful in informing the municipal child health services in the future.

## 2 METHODS

This paper reports data from semistructured interviews with six individual PHNs working at a CHC in a medium‐sized Norwegian city. The name of the city can be provided on request. A qualitative methodology was adopted to explore the PHNs' perceptions, knowledge and experiences. We used an inductive, data‐driven thematic analysis as it provides an accessible and theoretically flexible approach to analysing qualitative data that can be used across a range of epistemologies and research questions (Braun & Clarke, 2006). Thematic analysis is a widely used method in qualitative health‐ and well‐being research (Braun & Clarke, 2019), and is particularly relevant to applied research settings (Aquino et al., 2015).

The Norwegian Centre for Research Data evaluated and approved the study (26 March 2020, Reference 137889); the study was conducted in accordance with the Declaration of Helsinki. The study is reported following Standards for Reporting Qualitative Research (O'Brien et al., 2014; Supporting Information: SRQR Checklist).

### 2.1 Interview schedule

A semistructured interview schedule was developed by the authors (C. H., E. R. H. and N. C. Ø.) based on existing literature and the authors' prior experiences. Test interviews were conducted with colleagues. We included five open‐ended core questions, allowing the interviewer to explore issues raised by the interviewee. The questions intended to elicit the overall experience that PHNs have regarding nutritional guidance for parents as well as perceptions regarding their current knowledge (Table 1).

**Table 1:** Interview questions.

Interview questions
Can you tell me about your own background and how long you have worked as a public health nurse?
How do you feel about guiding parents of infants and toddlers on nutrition and diet at the child health centre?
What do you think about public health nurses' opportunities to provide parents with good and tailored information on food and nutrition at the child health centre today?
How often is diet a topic when following up parents of infants and toddlers at the child health centre?
What do you think about public health nurses' general knowledge when it comes to nutrition and importance of diet early in life?

### 2.2 Procedure

In beforehand there existed an agreement of intent between the university and the municipality involved in this study, which authorized the implementation of this project. The purpose of this agreement was to enhance collaboration between the municipality and the university within 15 specific areas, where one was related to research collaboration. The agreement was concluded at a high decision‐making level between the managements of the university and the municipality. In this municipality there was only one CHC. One of the authors (N. C. Ø.), who is the leader of the research team, contacted the leader of the CHC, who gave permission to approach and recruit PHNs to participate in the study. The first author (C. H.) presented the study and discussed what may be a good way forward in an initial planning meeting with the leader of the CHC and the team‐leader for the PHNs. As the interviews required a lot of time from the PHNs, it was important to take the management of the CHC's own priorities and wishes into account regarding the number of interviewees. It was agreed that the team leader selected 5–7 of a total of 12 PHNs to be interviewed based on variation in age and experience. This resulted in six PHNs agreeing to participate in the study. These nurses were invited to the study and provided with a participant information sheet by email by the first author (C. H.) well in advance of the interviews.

The semistructured interviews were conducted in January and February 2021 during the COVID‐19 pandemic by the first author (C. H.). No relationship was established between the interviewer and the interviewee before the commencement of the interviews. All interviews were taken once at the CHC during regular working hours with only the interviewer and interviewee present face to face, using a Zoom H1n Recorder. The consent form was signed by each interviewee before the interview. The interview began with C. H. introducing herself and confirming that the interviewee consented to recording of the interview, before briefly reviewing the aim of the interview. The anonymized interviews were transcribed verbatim in Norwegian by two trained research assistants. The transcripts were uploaded in NVivo 12 for analysis. As the interviews were conducted in Norwegian, the following analysis was performed in the original language. The quotes presented in this article were carefully translated to English from the original transcripts. A table with all the quotes that formed the basis of this analysis is included in the Supporting Information: Quotes.

### 2.3 Participants

A total of 12 PHNs were working at the CHC, all were females. Semistructured interviews were conducted with six of the nurses. Among these, the number of years working as a PHN varied between 3 and 40 years. However, all six PHNs had more than 10 years of experience working as a nurse. The current CHC covered the entire municipality, and all PHNs worked with families from both high and low socioeconomic areas. One of the PHNs had a special responsibility for working with refugee families, but all nurses regularly followed up immigrant families.

### 2.4 Data analysis

The analysis used a prescribed ‘step‐by‐step’ process following Braun and Clarke's (2006) guidelines of thematic analysis (Table 2). Although the process is described as ‘step‐by‐step’, the work was flexible and not linear. Through continuous discussions and revisions, a common understanding and agreement of themes and subthemes across the research team was achieved.

**Table 2:** Phases of thematic analysis.

The application of step‐by‐step thematic analysis based on Braun and Clarke (2006)	
1. Familiarizing yourself with your data	The digital interview recordings were transcribed verbatim by two trained research assistants. All three authors (C. H., E. R. H. and N. C. Ø.) read and re‐read the transcripts to become familiar with the data, noting down initial ideas
2. Generating initial codes	Initial codes were generated systematically across the entire data set (C. H.), collating data relevant to each code
3. Searching for themes	Codes were collated into potential themes and subthemes (C. H.), gathering all data relevant to each potential theme
4. Reviewing themes	All three authors (C. H., E. R. H. and N. C. Ø.) engaged in face‐to‐face discussion of the themes and subthemes to achieve a shared understanding of each theme and to ensure that the themes were applicable to the related coded extracts and the entire data set. Themes were reworked and subsequently validated across the data set. Finally, a thematic map was generated (C. H.)
5. Defining and naming themes	Themes were defined (C. H., E. R. H. and N. C. Ø.), and the overall story was drafted (C. H.)
6. Producing the report	Finally, all themes, codes and illustrative extracts were compiled together and refined by all three authors (C. H., E. R. H. and N. C. Ø.), linking the findings to previous literature and considering the broader impact of the findings

All three authors had a special interest in nutrition and eating behaviours during ‘the first 1000 days’—from conception to child age 2 years. However, the researchers still brought different perspectives to the analysis. Two of the authors are nutritionists (N. C. Ø. and E. R. H.). One of the nutritionists had in her career mainly worked as a researcher and research leader (N. C. Ø.), while the other had worked for several years as a clinical nutritionist in the hospital's children's department. The first author (C. H.) is a child psychiatrist with several years of clinical experience, including working with infants and toddlers having eating difficulties. As a multidisciplinary research team, we acknowledged how our individual understanding and academic/clinical experiences could influence the analysis. Through repeated discussions and critical interpretation of the data, we aimed to achieve a deeper understanding of the PHNs‘ experiences on nutritional guidance for parents of infants and toddlers.

## 3 RESULTS

**Figure 1:**
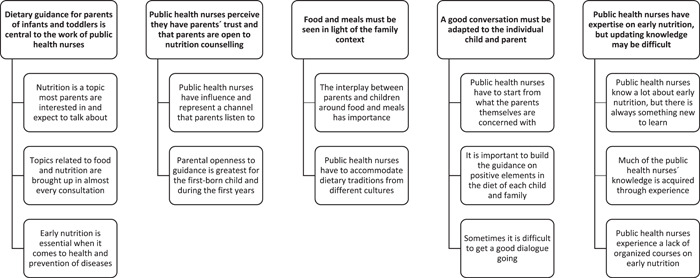
Themes and subthemes identified during thematic analysis.

The interviews lasted an average of 47 min (range 33–54 min). Across all interviews 5 themes and 13 sub‐themes were identified (see Figure 1). During thematic analysis, saturation was reached based on no further new information provided in the last interview. The themes and their respective sub‐themes are summarized below with illustrative quotes.

### 3.1 Theme I: Dietary guidance for parents of infants and toddlers is central to the work of the public health nurses

It became evident from the interviews that guidance on nutrition constitutes an important part of the PHNs' work. The thematic analysis revealed that the perceived importance of dietary guidance could be linked to three underlying themes:

#### 3.1.1 Nutrition is a topic most parents are interested in and expect to talk about

The majority of the PHNs reported that parents in general were very engaged and interested in their child's diet and nutrition. This was a topic on which parents frequently asked questions or sought advice, often concerned that the child should eat enough food of good quality to ensure growth and development. The PHNs further expressed that parents' involvement in their child's nutrition seemed linked to a desire to do what was best for their child.

*I think the parents are usually very interested. They want the best for their children and the best advice. (Interviewee 6)*

The PHNs also stated that the CHC was a place where parents expected to talk about their child's diet. This made it easy to bring up diet‐related topics during the consultations.

*In general, it's a harmless topic for everyone. And if there's anything they expect us to talk about, it's it. (Interviewee 5)*

#### 3.1.2 Topics related to food and nutrition are brought up in almost every consultation

All the PHNs reported spending a lot of time talking with parents about their child's diet and eating habits and nutritional guidance was included in nearly all consultations. Nutrition was described as a persistent and important topic from infancy onwards, covering different phases of eating development from breastfeeding, through introduction to solid food, and up to acceptance of the family's food. The PHNs further described nutrition counselling as a comprehensive topic covering more than ‘what to eat’, like supporting parents to achieve favourable feeding practices and mealtime routines as well as promoting food enjoyment.

*You nearly always ask about it… How is eating going? What do they eat? Only rarely is this not talked about. (Interviewee 1)*

*… It's more about food and meals, enjoyment of food and all that. Getting introduced to new foods, tastes, and smells… It's such a big topic when you start, it can actually take a whole consultation. (Interviewee 3)*

Some of the nurses held forward that the Norwegian national recommendations impose many tasks on the PHNs, and that in practice it was difficult to cover everything. Prioritizing was important—if something new was to be included in the consultation, something else had to be excluded. However, the PHNs spoke of nutrition as one of their main topics of high priority.

*… it's one of the things we have at the top of our list, along with everything else we're going to talk about. But it's brought up every time, actually. (Interviewee 4)*

Although nutritional guidance for parents was perceived as a large and comprehensive topic, the PHNs believed they had the necessary flexibility to attend to the individual parent's needs. Albeit several of the nurses claimed that time was a limited resource, they had the option of offering parents a new appointment or phone call if they found it necessary.

*Parents may say “I'm wondering a bit about my child's diet, may I have a new appointment, or can you call me?” Then we schedule an extra appointment or call the mother… they don't always need to come here. (Interviewee 2)*

#### 3.1.3 Early nutrition is essential when it comes to health and prevention of diseases

The importance of early nutrition was linked to the child's health and the prevention of NCDs, particularly overweight and obesity. The nurses talked about how children's early diet was fundamental for later health and well‐being.

*Well ‐ diet is linked to many lifestyle diseases. So, from a prevention point of view, it is extremely important. (Interviewee 5)*

Some of the nurses expressed that being able to work with early prevention was an important part of their commitment and increased their own motivation.

*That's why I wanted to become a public health nurse,… you can prevent obesity or heart disease, diabetes… you can influence the parents early on, lead them in a healthy direction… (Interviewee 2)*

### 3.2 Theme II: Public health nurses perceive they have parents' trust and that parents are open to nutrition counselling

#### 3.2.1 Public health nurses have influence and represent a channel that parents listen to

The majority of the PHNs felt that their opportunity to give parents dietary guidance at the CHC was good. As all children are regularly seen at CHCs, the PHNs get to know families well. The CHC was perceived as a natural place for parents to seek advice about their child's health, growth, and development, and the PHNs adapted the information they were to convey to the individual family.

*Yes, the opportunity is great because we follow them over time.//You have to know from one time to the next that for this particular family I have to talk about this very thing because I know that, for example, they don't use milk products. (Interviewee 3)*

#### 3.2.2 Parental openness to guidance is greatest for the first‐born child and during the first year

Although the PHNs experienced that early nutrition was a topic that parents in general were engaged with, they also reported that parental commitment and openness to nutritional guidance could vary over time. The child's first year of life was described as a period in which parents were particularly open to guidance. Parents were often concerned about doing ‘everything right’, especially in the transition phase when solid food was to be introduced. Questions on what kind of food the child should be introduced to and how much they were expected to eat were frequent.

*…it's a big part of the first year at least. Some have a lot of questions and… or, most have a lot of questions, and especially regarding the transition from milk to solid food and things like that. (Interviewee 1)*

As the child grew older, the PHNs often experienced that parents' interest in the child's diet decreased, and that parents were not as receptive to guidance. The family's dietary habits seemed established and thus more difficult to influence.

*…but perhaps when the child turns two years old or older, they may not be as open for guidance, so you can notice a bit of a distance if you try to regulate… (Interviewee 2)*

This also applied to parents with more than one child. Parents with older children were perceived to be less open to new input from the PHNs compared to first‐time parents.

*First‐time parents can be very receptive and very ignorant simultaneously.//…at the same time, we have those who have several children and don't want to listen to anything. And a bit like “Yes, you say so, but I do what I want”, a bit like that. (Interviewee 3)*

### 3.3 Theme III: Food and meals must be seen in light of the family context

#### 3.3.1 The interplay between parents and children around food and meals has importance

The PHNs reported that difficulties related to the child's nutrition could affect the emotional climate in the family. Early feeding difficulties in premature infants could contribute to persistent anxiety and worrying in parents, leading to continued excessive use of pressure to eat over time. When children were picky, were having poor appetite or refused to eat, meals could turn into a fight.

*…regarding those babies who started life with… being premature or having early difficulties, where it is so firmly rooted in the parents. The importance of feeding the child during the first weeks and months in hospital. And then it ends in trouble when the child is one year old, three years old, five years old. It is still there, and the parents talk about it as if it happened yesterday. (Interviewee 3)*

The PHNs considered it important to promote enjoyment of food and beneficial eating habits. They experienced that some young parents strove to establish good mealtime routines. In other families, children had been given too much leeway and parents struggled to cope with their parental responsibility during mealtimes.

*Yes, I've become more concerned with this as years go by, the importance of food enjoyment and that meals become something cozy and nice. I have experienced coming home to young families who don't even have a dining table in their home. Then I think “Oh, my goodness, they're eating in the sofa watching TV”. I think it is so sad, they miss out on so much. (Interviewee 5)*

#### 3.3.2 Public health nurses have to accommodate dietary traditions from different cultures

Culture‐specific traditions related to infant nutrition seemed to be a recurring theme in PHNs' dietary guidance. Sometimes it was challenging when traditional practices in child dietary care were opposed to nutritional guidance based on the Norwegian recommendations. Although the PHNs were obliged to follow the official guidelines, they acknowledged that this could be a conflict of loyalty for the parents.

*And then it's about traditions. If you come from other cultures and countries, then it can be difficult, right, because there they have done this and that. But in Norway we do not recommend this. And then they must choose; should they listen to me, or should they listen to themselves and where they come from? (Interviewee 2)*

Parents from other cultures could further have a different opinion regarding the child's weight, considering the child as being underweight when it was of normal weight. The nurses saw this in the context of coming from a country where food could be scarce.

*And… many in the group I work with, they… when the children are of normal weight, they think they are so thin, you know ‐ they want them to be more… robust. (Interviewee 4)*

At the same time, some of the PHNs felt that many immigrants often had a rich food culture and were more engaged in cooking and bringing the family together around the meals than ethnically Norwegian families. Most immigrants were recognized to be open and receptive to nutritional guidance. However, it was perceived as especially important to ask detailed, concrete, and accurate questions to reach this group.

*I spend a lot of time on it… but it is not because there are necessarily a lot of problems around it, more because… I often have to ask such detailed questions to get…//my impression is that these cultures are characterized by more cooking from scratch and that the whole family gathers around it. (Interviewee 4)*

### 3.4 Theme IV: A good conversation must be adapted to the individual child and parent

#### 3.4.1 Public health nurses have to start from what the parents themselves are concerned with

Based on the interviews, it was consistent that the PHNs emphasized what the parents themselves were concerned with for the moment in their consultations. The PHNs could have planned what was to be brought forward, but if something else was more urgent to the parents this was given priority. The PHNs focused on parents' urgent needs to ensure dialogue and contact and strove to tailor topics and guidance to circumstances. To talk about something parents were not interested in was perceived as creating distance and not leading to progress. If they missed out on important topics, they would normally schedule a new appointment.

*…it's really up to the parents…//I may have planned it, but then suddenly they are concerned with something different… and then we might only have two minutes at the end. So, it varies a lot… but it has to be, in a way… it's a little bit what the parents are concerned with and then… you have to guide from there. (Interviewee 1)*

*There is no point in me talking and informing about things if it is not something they are genuinely concerned with. So, if there is something else that is very important to them, then they will not listen to what else I say. So, then we have the opportunity to bring it up… that is on a later occasion. (Interviewee 4)*

#### 3.4.2 It is important to build the guidance on positive elements in the diet of each child and family

When giving guidance to parents on nutrition, it was important to make the information clear and simple to promote motivation and reinforce parental self‐confidence. It was further perceived as important to build on existing positive elements in the diet of each individual child and family.

*It doesn't have to be such a fixed framework. I feel that if someone is like that when giving advice, then they become a somewhat rigid public health nurse… or, yes, right… you have to make room for individual differences and what kind of foods children like. (Interviewee 2)*

The PHNs perceived that some parents were very concerned about doing everything correctly and afraid of making mistakes. They often asked for concrete answers and clear‐cut advice, which could come into conflict with the PHN's way of guiding. The PHNs sought to enhance parenting skills by emphasizing parental intuition and flexibility in line with the child's specific needs to build parental trust in own judgement. They further strove to make nutrition less complicated by focusing on ‘what's good enough’.

*I do understand it when it's the first child, …and you're perhaps used to having full control and things, like having a recipe to follow and so on… but then you also have to learn that children are different… (Interviewee 1)*

A few parents did not have the energy or strength for neither meal planning nor cooking, for example, due to economic worries or mental illness. One of the PHNs talked about how it was important not to impose even greater burdens on these mothers in form of unmanageable demands and expectations:

*…and then I think that you have to be careful with what you kind of add to them in terms of expectations in addition to everything else they are struggling with. So, in some ways you have to find a balance there… at least find what's good enough, and not make them feel even more guilty about everything they can't afford, or have to reach for, or endure and so on… (Interview 4)*

#### 3.4.3 Sometimes it is difficult to get a good dialogue going

Although nutrition was a topic most parents were interested in and which was usually unproblematic to bring up, the PHNs sometimes found that the dialogue could be challenging. Occasionally, the conversation did not turn out as planned, and parents missed out on what was being communicated. The PHNs seemed to accept that they could not impose information on parents and that ultimately it was the parents themselves who decided.

*But there are always some who choose their own ways of doing things and who don't easily follow the advice they get, but that is a choice they make. And well, we can't go home to them to prepare dinner. (Interviewee 6)*

The PHNs also reported that some parents found it offensive to receive comments or suggestions for improvements in their diet. This could lead the conversation to get stuck with parent and PHN caught in defensive positions.

*They say they have read a lot about something and are going to reprimand me for this and that, but they have misunderstood, and I don't reach them with the details…//If you first get stuck, it's words against words… (Interview 3)*

A striking finding across all the interviews was how difficult the PHNs perceived it was to talk about nutrition related to overweight and obesity. Overweight and obesity was a topic that received particular focus at the CHC. Still, all the PHNs expressed that raising this topic with parents was challenging. Providing guidance on the child's overweight, while at the same time maintaining the relationship with the parents, was experienced as a difficult balancing act. The PHN's perceived that parents often had their own experiences with overweight that were awakened when the topic was brought forward, and that this could provoke aggression.

*Regarding overweight and such, it is not always easy to guide on that, or they are not so receptive for guidance.//It's not always that it… is so well received, not everyone finds it easy to talk about or receive guidance on it. Yes, it often is… that it can be difficult. (Interviewee 1)*

*In a way, it's a harmless topic… Until we get to those, I don't know what to call it, I might call it the stigmas, especially in relation to obesity. Where we can get a lot of aggression and hurt feelings because it is so much into the history of the parents. (Interviewee 3)*

### 3.5 Theme V: Public health nurses have expertise on early nutrition, but updating knowledge may be difficult

#### 3.5.1 Public health nurses know a lot about early nutrition, but there is always something new to learn

When the PHNs described their own knowledge of early nutrition, they expressed a certain duplicity. All declared that their general knowledge on this topic was good. They were familiar with the national recommendations and how to use these guidelines in clinical practice. In addition; as a group, they had extensive expertise, where some PHNs contributed with long clinical experience, while newly qualified PHNs contributed with more ‘up‐to‐date’ knowledge.

*I think we know quite a lot…//And overall, combined we have much and long experience. Several of us have worked for many years… And new nurses come in and bring new things with them, so I think our combined knowledge is quite large. (Interviewee 4)*

At the same time, the nurses added that it could be challenging to keep up to date on early nutrition due to continuous supply of new knowledge. Parents constantly came up with new questions, and professional deepening required time and personal interest. Occasionally, this could give a feeling of falling short.

*Obviously, different things pop up that are new to us, where we don't have full control.//And I find it difficult because I don't always have enough knowledge, but then you know that there are some good websites that you can read. But then I feel like “Ugh, is this good enough?” (Interviewee 5)*

#### 3.5.2 Much of the public health nurses' knowledge is acquired through experience

All the nurses described that nutrition had been a topic in their education, but how comprehensive they remembered this to be differed. While two of the nurses recalled that nutrition had been an important topic, the remaining nurses seemed unsure of how much nutritional education they had actually received.

*Yes, it probably was a topic, but I don't remember that we had very much about it.//It hasn't been that long since I graduated, but it wasn't that much anyway… we probably had a bit… curves and all that, but… (Interviewee 1)*

In contrast, all the nurses declared that nutrition was a major topic at the CHC, and the majority of the nurses said they had acquired most of their knowledge through experience.

*It was, but… maybe not such a huge focus on it? I really think I've learned a lot more after I finished my education. (Interview 2)*

#### 3.5.3 Public health nurses experience a lack of organized courses on early nutrition

Although the PHNs stated that early nutrition was a central topic in their clinical work, this was not reflected in the offering of courses or further education. All the nurses said it had been a long time since the Norwegian Nurses' Association last organized a course on early nutrition. In contrast, courses organized by commercial actors used to be offered regularly before the Covid‐19 pandemic. In addition, nutrition could occasionally be a topic of internal teaching.

*No, not that much about it (courses on nutrition). I don't think so, not that I can remember during the last few years at least. (Interviewee 6)*

Some of the nurses expressed a wish for more courses on early nutrition, as this would contribute to their own professional development.

*I think we could have had even more courses on nutrition…//… it is very useful to have input from courses, because that is what makes one… develop then. (Interviewee 2)*

## 4 DISCUSSION

This study aims to investigate PHNs' perspectives and experiences regarding nutritional guidance for parents of infants and toddlers and how they perceive their own knowledge of the topic. We wanted to identify potential facilitators, barriers and needs to provide good guidance and appropriate care that could be useful in informing the municipal child health services in the future.

Our findings highlight how important and essential the PHNs consider nutrition counselling to be in their daily work. The official Norwegian recommendations for child health services have nutrition as a persistent theme from infancy throughout toddlerhood (Norwegian Health Directorate, 2017). We found no evidence of contradiction between the guidelines' requirements and the PHNs' perceptions of the importance of early nutrition. Neither did the PHNs report that nutrition counselling came into conflict with other topics of interest. Rather, the central role and high priority given nutrition counselling was underpinned by early diet being a topic of large importance to parents and of formative significance for children's future health and wellbeing. Although other issues could sometimes be more pressing and limit the amount of time available, the PHNs seemed to have sufficient flexibility in their workload to offer a new appointment if necessary. Our findings are in line with previous studies, finding that dietary guidance is considered by PHNs to be an important topic in consultations with parents (Holmberg Fagerlund et al., 2016; Ilmonen et al., 2012; Kim & Choue, 2009).

In addition, this study reveals how PHNs feel they have parents' trust regarding nutrition counselling and that parents consider the CHC a natural place to raise questions about diet and eating behaviours. Previous studies confirm that PHNs in general have trust among their users (Boelsma et al., 2021; Clancy, 2007; Clancy & Svensson, 2010; Holmberg Fagerlund et al., 2019), although some studies find that trust may vary (Henström et al., 2020). Our findings further emphasize the importance of getting to know children and parents over time to build mutual trust. An established relationship enables the PHN to raise personal issues related to diet and eating behaviour and provide nutritional support in a more customized way. The relationship between PHNs and their clients has been explored by observations in a previous Norwegian qualitative study (Clancy & Svensson, 2010). This study describes how both nurses and clients picked up where they had left off at their previous visit and ended the consultations by referring to their next appointment. This led to a type of continuity where consultations did not appear as isolated incidents, but as events on a continuum. The authors concluded that continuity and trust in services seemed paramount to the service users' satisfaction. Our results confirm these findings, showing that sustained relationships with clients facilitate the possibilities for the PHNs to provide tailored counselling.

Our findings point to the first 1000 days as a window of opportunity for nutritional counselling. In line with previous research (Henström et al., 2020), parents were perceived as more receptive to guidance in this phase. Parents in this period are particularly concerned about their child's nutrition, and many parents experience worries related to diet or eating behaviours (Almqvist‐Tangen et al., 2017; Henström et al., 2020; Norlyk et al., 2019). Studies exploring PHNs' experiences have documented that counselling about food and feeding practices typically becomes challenging when the child reaches weaning age and begins to eat the same food as the rest of the family (Ersson et al., 2021; Holmberg Fagerlund et al., 2016). Our results further support the importance of reaching first‐time parents. Being a parent during the child's first year can be experienced as overwhelming (Nystrom & Ohrling, 2004). The transition to parenthood involves major lifestyle changes, and first‐time parents may face difficulties and lack self‐efficacy in fulfilling the expectations of their new roles (Entsieh & Hallström, 2016; Norlyk et al., 2019). Receiving professional support is a vital component of developing parental practices (Holmberg Fagerlund et al., 2019). Overall, the results of our study underpin the need for good nutritional care during this period. As families today live further away from relatives and lack of social networks is an issue of increasing importance, the role of the PHNs is becoming more important in parents' lives (Ersson et al., 2021).

The current study further illuminates the broad focus the PHNs apply in their guidance on diet and feeding practices and their concern with how food and meals form part of family life. If a child ate poorly this could affect the emotional climate of the whole family. Feeding difficulties are perceived as very stressful for parents, even when the feeding difficulties are not accompanied by nutritional disorders or organic disease (Goday et al., 2019; Kerzner et al., 2015). The fact that the difficulties are not reflected in weight or height does not prevent parents from experiencing levels of anxiety and stress that may exceed those generated by organic diseases (Fernández de Valderrama Rodríguez et al., 2022). This can give rise to vicious circles, where parents' elevated levels of stress and anxiety increase their use of controlling feeding practices, which in turn may lead to more food refusal in the child (Jansen et al., 2018). Negative experiences related to feeding may also lead to avoidance of joint family meals. Sharing meals as a family is associated with more nutrient‐dense food intake, less fussy eating, and higher levels of food enjoyment as well as health benefits in infants and toddlers (Verhage et al., 2018). Family dietary and lifestyle practices may have long‐term positive or negative effects, as health behaviours established in childhood tend to be maintained during later life with tracking over time (Montaño et al., 2015). Guiding and helping families to positive and health‐promoting family meals is therefore an important, yet potentially challenging, task for the PHNs.

Also, the families' cultural background influenced the PHMs' nutrition counselling. The PHNs strived to adapt care to accommodate religious, cultural and linguistic differences if this was compatible with the official Norwegian recommendations. However, language barriers could make the dialogue difficult. The language had to be simple and concrete, and visual material like pictures was frequently used. A previous Norwegian qualitative study on nutrition counselling towards immigrant mothers reported similar findings. They described how PHNs adopted simplistic communication styles that were considered as more effective, but which made the PHNs feel uneasy. Some created their own visual communication tool to overcome challenges arising from language barriers (Holmberg Fagerlund et al., 2016). The PHNs in our study further reported cultural differences regarding parents' perceptions of their child's weight. Immigrant parents often worried for their child being too thin, even if the percentile scheme indicated normal weight. A previous Dutch study has described how chubby babies were considered to be healthy babies (Boelsma et al., 2021). A previous Norwegian study reported how parental concerns regarding overweight seldom occurred among immigrant parents, and that some parents rather were proud of their child being overweight (Holmberg Fagerlund et al., 2016). A Swedish study found an increased risk of being overweight among offspring of immigrants consistent with findings from other European studies (Besharat Pour et al., 2014). Overall, our study confirms previous findings and points to the need for a culturally sensitive approach when counselling immigrant parents on early nutrition.

The PHNs placed great emphasis on the importance of dialogue when counselling parents. The conversation had to be adapted to what was pressing to the parents and to the needs of each individual client. This required good and varied communication skills. The PHNs applied empowerment strategies by building the conversation on existing positive elements to enhance parenting skills and confidence. To strengthen empowerment, the dialogue should focus on the resources and knowledge the families already possess, rather than on what they lack or need (Håkansson et al., 2019). The PHNs further guided on promoting responsive feeding, characterized by caregiver guidance and recognition of the child's cues of hunger and satiety (Hurley et al., 2011). Several studies have found correlations between responsive feeding practices and positive impacts on children's dietary patterns and weight (Daniels et al., 2015; Hohman et al., 2017; Savage et al., 2016), and elements of responsive feeding are included in the official Norwegian recommendations for child health services (Norwegian Health Directorate, 2017). However, the PHNs experienced that attempts to make parents more aware of the child's signals to better understand the child's needs often conflicted with parents‘ desire for concrete answers to their questions. A previous study found that parents of infants perceived messages like ‘try and see what works’ as difficult to interpret and put in practice (Henström et al., 2020). Our findings may indicate that guidance on parent–child interplay requires more available time and opportunities for direct observations than what PHNs have at their disposal.

It was a consistent experience among the PHNs that the dialogue with parents could occasionally be difficult. Some parents found it offensive to receive suggestions for improving their diet. Previous research has shown that parents may experience nutrition counselling as intrusive, especially if their diet preferences are not in line with the diet recommendation presented (Holmberg Fagerlund et al., 2019). In our study, the dialogue could further be difficult in relation to sensitive topics, particularly related to overweight and obesity. Previous studies confirm that parents may react with negative feelings when the child's weight status is brought up (Ames et al., 2020; Regber et al., 2013). A Swedish qualitative study among PHNs found that weight‐related discussions were facilitated by building and maintaining trust with parents. However, the PHNs were reluctant to address children's weights if this could compromise parents' trust. The nurses identified a lack of sufficient knowledge about what to offer the family and a lack of confidence in their communication skills as additional barriers (Sjunnestrand et al., 2019). Taken together, our study confirms previous findings and underlines the great importance of dialogue in nutritional guidance and the considerable demands it places on good and varied communication skills.

Lastly, our findings point out that even though nutrition counselling stands out as an important, multifaceted and central part of the PHNs' work at the CHC, this is to a lesser extent reflected in their education and course offerings. The PHNs were unsure of the extent of nutritional education they had received in their education and expressed that a large part of their knowledge was acquired through experience. They further reported that although there was a continuous supply of new knowledge in this area, the offering of continuing education or courses on the topic was sparse even before the Covid 19 pandemic. The Norwegian Regulations on national guidelines for PHN education states that public health nurses must possess the competence to ‘independently provide guidance on nutrition, nutritional challenges, breastfeeding and growth and to refer further if necessary’. However, it is not defined which nutrition‐related topics the study plan is supposed to cover, and what different educational institutions offer in terms of nutritional education may vary. Given the influential task laid upon the PHNs regarding conveying nutritional knowledge to parents in an important phase, there seems to be a discrepancy between how nutrition is prioritized in the education of PHNs and what they encounter in clinical practice.

### 4.1 Strengths and limitations

There are several strengths with this study. The PHNs interviewed had different experience before they became PHN and varied duration as PHN, providing different perspectives and insights. The interviewer was skilled in dialogues and experienced within early childhood care. In addition, the authors had different work experience, providing broader perspectives to the interpretation of the findings. Our findings are in line with previous research from other countries.

There are also limitations to consider. The existing agreement of intent between the university and the current municipality may have influenced the individual nurse's consent to participate in the study. Still, it is less likely that this agreement had any significance for what was communicated in the individual interviews and the results as such. The interviewees worked in the same unit which may have led to a homogeneity in their reflections and responses. However, they worked quite independently, and were responsible for their own users. Another limitation is that the leader was asked to select the interviewees, which means that we cannot exclude selection bias. The authors' professional background may be a cause of confirmation bias and an increased reported nutritional focus. Lastly, our findings may not be generalizable to other regions. Although the activities of the Norwegian municipal health centres are anchored in a common national guideline, there may be differences linked to local conditions and our findings may therefore not be representative for the whole country.

## 5 CONCLUSION

Our study underpins the importance of the first 1000 days as a window of opportunity for nutritional counselling of parents. In this developmentally important phase, parents are perceived as particularly open to guidance and their need for good nutritional care is at its highest. Guidance on diet and nutrition is perceived by PHNs as a core activity and constitutes a large part of their communication with parents of infants and toddlers. The PHNs feel they have parents' trust when guiding on dietary issues and strive to tailor their guidance to meet individual demands and ensure dialogue and contact. However, conversations around issues such as childhood overweight and obesity are perceived as sensitive and difficult. Our results underline the importance of dialogue in nutritional guidance and the considerable demands placed on good and varied communication skills. Our findings also suggest that there seems to be a discrepancy between how nutrition is prioritized in the education of PHNs and what they encounter in clinical practice. This should be given more attention given the unique position PHNs have regarding parents' trust and possibility to promote healthy eating and long‐term health.

## AUTHOR CONTRIBUTIONS

All three authors (Christine Helle, Elisabet Rudjord Hillesund and Nina Cecilie Øverby) provided substantial contributions to the conception and design of this study. Christine Helle collected the data and drafted the paper. All three authors contributed to the interpretation of data. Elisabet Rudjord Hillesund and Nina Cecilie Øverby critically reviewed the manuscript. All the named authors have made an active contribution to the final version of the paper and approved the submitted manuscript for publication.

## CONFLICT OF INTEREST STATEMENT

The authors declare no conflict of interest.

## Supporting information

Supporting information.

Supporting information.

## Data Availability

Data are available in the supplementary material.
