# Supplementary material for: A qualitative study of public health nurses' perspectives and experiences on nutritional guidance for parents of infants and toddlers
Source: Matern Child Nutr. 2023 Jul 13;20(Suppl 2):e13546. doi: 10.1111/mcn.13546 (PMC10765362; doi:10.1111/mcn.13546)
Supplement: Supplementary file 2 — Supporting information. [file MCN-20-e13546-s002.docx]

# **Quotes**

| **Theme I** | **Subthemes** | **Sample quotes** |
| --- | --- | --- |
| **Dietary guidance for parents of infants and toddlers is central to the work of the public health nurses** | Nutrition is a topic most parents are interested in and expect to talk about at the child health centre | *"I think parents are usually very interested. They want the best for their children and the best advice." (Interviewee 6)*  *"In general, it's a harmless topic for everyone. And if there's anything they expect us to talk about, it's it" (Interviewee 5)* |
|  | Topics related to food and nutrition are given priority and brought up in almost every consultation | *"You nearly always ask about it... How is eating going? What do they eat? Only rarely this is not talked about." (Interviewee 1)*  *"It's a big topic when we start talking more about diet - or I don't like the word 'diet' because it's not really that. It's more about food and meals, enjoyment of food and all that. Getting introduced to new foods, tastes, and smells... It's such a big topic when you start, it can actually take a whole consultation." (Interviewee 3)*  *"... it's one of the things we have at the top of our list, along with everything else we're going to talk about. But it's brought up every time, actually." (Interviewee 4)*  *"Parents may say “I'm wondering a bit about my child`s diet, may I have a new appointment, or can you call me?" Then we schedule an extra appointment or call the mother... they don't always need to come here" (Interviewee 2)* |
|  | Food and nutrition are essential when it comes to health and early prevention of diseases | *"Trying to say very early on that the food you give the child right from the start lays the foundation for the rest of the child's life. I expect parents to want their children to eat healthy and not having weight problems as adults." (Interviewee 2)*  *"Well - diet is linked to many lifestyle diseases. So, from a prevention point of view, it is extremely important." (Interviewee 5)*  *"That's why I wanted to become a public health nurse, ... you can prevent obesity or heart disease, diabetes... you can influence the parents early on, lead them in a healthy direction..." (Interviewee 2)*  *"So, I think that it is good information to share, and important. Yes, absolutely important, and perhaps even more important than before.” (Interviewee 6)* |

| **Theme II** | **Subthemes** | **Sample quotes** |
| --- | --- | --- |
| **Public health nurses perceive they have parents´ trust, and that parents on general are engaged and open to nutrition counselling** | Public health nurses have influence and represent a channel that parents listen to | *"I think so. That we are... a channel that they want to listen to. And the vast majority visit the child health centre... there are very few who are not here... so" (Interviewee 2)*  *"Yes, the opportunity is great because we follow them over time. // You have to know from one time to the next that for this particular family I have to talk about this very thing because I know that, for example, they don't use milk products" (Interviewee 3)* |
|  | Nutrition is a topic parents on general are engaged in, but interest and openness to guidance is greatest for the first-born child and during the first years | *“...it's a big part of the first year at least. So... some have a lot of questions and... or, most have a lot of questions, and especially regarding the transition from milk to solid food and things like that." (Interviewee 1)*  *"I find that this is something most parents are concerned about when the children are young. When they are less than one year old, especially.” (Interviewee 5)*  *«...when they get a bit older, then, it is very variable how receptive the parents are, or how much they want ... / / They are not interested, or they have found their ways. / /... yes, there is a big difference between the first year maybe, and when they are a bit older... maybe it can be easier when they are younger." (Interviewee 1)*  *«...but perhaps when the child turns two years old or older, they may not be as open for guidance, that is you can notice a bit of a distance if you try to regulate..." (Interviewee 2)*  *"I feel they are very open and seeking advice... and accept... guidance, especially first-time parents are very interested, and mostly I feel that they are very interested in dietary guidance.../ / I think there is a big difference, when they have done this before... they ask, but then they know a lot, but we repeat anyway..." (Interview 2)*  *"First-time parents can be very receptive and very ignorant simultaneously. / / …at the same time, we have those who have several children and don't want to listen to anything. And a bit like "Yes, you say so, but I do what I want", a bit like that."* |

| **Theme III** | **Subthemes** | **Sample quotes** |
| --- | --- | --- |
| **Food and meals must be seen and understood enlightened by the family context** | The interplay between parents and children around food and meals has importance | *«...and it really affects both the child and the whole family if a small child.. for instance, eats poorly..." (Interviewee 2)*  *«...regarding those babies who started life with ..being born prematurely or having early difficulties, where it is so firmly rooted in the parents. The importance of feeding the child during the first weeks and months in hospital. And then it ends in trouble when the child is one year old, three years old, five years old. It is still there, and the parents talk about it as if it happened yesterday" (Interviewee 3)*  *"…and the children are very selective, and they struggle with meals turning into a battle where they run after the children with slices of bread in the living room, feeling that this is not fun at all." (Interviewee 5)*  *"Yes, I've become more concerned with this as years go by, the importance of food enjoyment and that meals become something cozy and nice. I have experienced coming home to young families who don't even have a dining table in their home. Then I think "Oh, my goodness, they're eating in the sofa watching TV". I think it is so sad, they miss out on so much." (Interviewee 5)* |
|  | Public health nurses have to accommodate eating habits and dietary traditions from different cultures | *"Yes, they often pick up a lot of information from their home country, and that's fine, but there may be quite different recommendations than the ones we have. / / So, you can be a bit surprised from time to time regarding what they have given to the children” (Interviewee 1)*  *“And then it’s about traditions. If you come from other cultures and countries, then it can be difficult, right, because there they have done this and that. But in Norway we do not recommend this and that. And then they must choose; should they listen to me, or should they listen to themselves and where they come from? This is a recurring problem" (Interview 2)*  *" ...In some cultures, they think that "the kid here looks so thin... and everyone in the family says that there must be something wrong»... / / So then... I have occasionally printed out the weight-curves, then they can show the whole family ... "look here - completely on average and fine". I try to reassure them in that, because they are distressed by the feedback from the family. But it's probably some cultures that want fat children because they have to survive..." (Interviewee 2)*  *"And... many in the group I work with, they... when the children are of normal weight, they think they are so thin, you know - they want them to be more… robust" (Interviewee 4)*  *"I spend a lot of time on this... but it is not because there are necessarily a lot of problems around it, more because... I often must ask very detailed questions to get... / / ...my impression is that these cultures are characterized by more cooking from scratch and that the whole family gathers around it." (Interviewee 4)* |

| **Theme IV** | **Subthemes** | **Sample quotes** |
| --- | --- | --- |
| **It is essential to adapt the dialogue to the individual child and parent to initiate a good conversation** | To reach the parents, public health nurses must start from what the parents themselves are concerned with | *«...it's really up to the parents... / / I may have planned it, but then suddenly they are concerned with something else... and then we might only have two minutes at the end. So it varies a lot... but it has to be, in a way... it's a little bit what the parents are concerned with and then... you have to guide from there" (Interviewee 1)*  *"But it is adapted from one time to the next and to each individual, because the starting point is the parents' needs." (Interviewee 3)*  *"There is no point in me talking and informing about things if it is not something they are genuinely concerned with. So, if there is something else that is very important to them, then they will not listen to what else I say. So, then we have the opportunity to bring it up ... that is on a later occasion." (Interviewee 4)* |
|  | It is important to make dietary guidance understandable and easily accessible, focusing on the child`s and the family`s individual needs. | *"It doesn't have to be such a fixed framework. I feel that if someone is like that when giving advice, then they become a somewhat rigid public health nurse... or, yes, right... you have to make room for individual differences and what kind of foods children like." (Interviewee 2)*  *"I do understand it when it's the first child, …and you're perhaps used to having full control and things, like having a recipe to follow and so on... but then you also have to learn that children are different…" (Interviewee 1)*  *"They think it's good to be able to talk about what really matters. How easy things can actually be done in relation to diet and nutrition. Especially those who have to do everything correctly, then I give a lot of feedback that it seems very complicated to do everything right." (Interviewee 3)*  *"...and then I think that you have to be careful with what you kind of add to them in terms of expectations in addition to everything else they are struggling with. So, in some ways you have to find a balance there... at least find what's good enough, and not make them feel even more guilty about everything they can't afford, or have to reach for, or endure and so on..." (Interview 4)* |
|  | Sometimes it is difficult to get a good dialogue going | *"I may get to say a lot about what I know I'm supposed to say. But then it's not always turning into this dialogue where I get to.. well, "did you really get this with you, or did it drown in everything else?". / / ... later one can repeat the same thing and then "oh, they haven't done what we recommended", also "oh no, they weren't there”. Then I didn't reach them with my guidance". (Interviewee 5)*  *".. I may say what I want here, but what they end up doing at home, that's up to them... isn't it, so sometimes you talk on deaf ears. But hopefully something got through then" (Interviewee 2)*  *"But there are always some who choose their own ways of doing things and who don't easily follow the advice they get, but that is a choice they make. And well, we can't go home to them to prepare dinner" (Interviewee 6)*  *"After all, it is the parents who 'run the shop', if you notice that they give totally unhealthy things... and comment on it... then there is not always a happy atmosphere, so to speak" (interviewee 2)*  *"They say they have read a lot about something and are going to reprimand me for this and that, but they have misunderstood, and I don't reach them with the details...// If you first get stuck, it´s words against words...” (Interview 3)*  *"Regarding overweight and such, it is not always easy to guide on that, or they are not so receptive for guidance. / / It's not always that it... is so well received, not everyone finds it easy to talk about or receive guidance on it. Yes, it often is... that it can be difficult." (Interviewee 1)*  *"In a way, it's a harmless topic... Until we get to those, I don't know what to call it, I might call it the stigmas, especially in relation to obesity. Where we can get a lot of aggression and hurt feelings because it is so much into the history of the parents." (Interviewee 3)*  *"Bringing up nutrition as a topic is usually not difficult. It is when they are overweight, then it becomes difficult." (Interview 5)* |

| **Theme V** | **Subthemes** | **Sample quotes** |
| --- | --- | --- |
| **Public health nurses have expertise on the subject, however updating knowledge may be difficult** | Public health nurses know a lot about early nutrition, but there is always something new to learn | *"I think we know quite a lot ... / / And overall, combined we have much and long experience. Several of us have worked for many years ... And new nurses come in and bring new things with them, so I think our combined knowledge is quite large." (Interviewee 4)*  *"I think, we could certainly update ourselves even more... well, I believe everyone has a use for that in all types of professions. That one may become outdated. So.. one will never be good enough, really. / / I think it (the knowledge) is good, but can certainly be improved, I would say…" (Interviewee 2)*  *"Obviously, different things pop up that are new to us, where we don't have full control. / / And I find it difficult because I don't always have enough knowledge, but then you know that there are some good websites that you can read. But then I feel like "Ugh, is this good enough?" (Interviewee 5)* |
|  | Much of the public health nurses´ knowledge is acquired through experience | *"Yes, it probably was a topic, but I don't remember that we had very much about it. / / It hasn't been that long since I graduated, but it wasn't that much anyway...we probably had a bit... curves and all that but..." (Interviewee 1)*  *"There was some focus on it of course, and we probably had... can't remember if we had a nutritionist who taught us about it, I can't really believe it actually... could certainly have been more" (Interviewee 2)*  *"I feel maybe a lot of that I have with me from... my own experiences from... reading me up." (Interviewee 1)*  *"It was, but... maybe not such a huge focus on it? I really think I've learned a lot more after I finished my education" (Interview 2)*  *"Yes, that is - area of expertise is because I have so much experience" (Interviewee 4)* |
|  | There is a lack of organized courses on early nutrition | *"It's mostly like that there... some is through Nestlé, for example, they have courses like that... / / Not so much beyond that really... I think? But a two-hour course or so...just very short...yes" (Interviewee 1)*  *“No, not that much about it. I don't think so, not that I can remember during the last few years at least." (Interviewee 6)*  *"I think we could have had even more courses on nutrition... // ... it is very useful to have input from courses, because that is what makes one... develop then." (Interviewee 2)*  *"But there really aren't many who offer courses on this, and we don't think that's quite alright." (Interviewee 5)* |
